# Symbolic representation by a two-dimensional matrix for profiling comparative animal behavior

**DOI:** 10.3389/fpsyg.2024.1450754

**Published:** 2024-11-22

**Authors:** Louis N. Irwin

**Affiliations:** Department of Biological Sciences, University of Texas at El Paso, El Paso, TX, United States

**Keywords:** subjective experience, proxy behavior, animal phenomenology, behavioral profiles, evolution of cognition, consciousness

## Abstract

The growing view that consciousness is widespread, multimodal, and evolutionarily non-linear in complexity across the animal kingdom has given rise recently to a variety of strategies for representing the heterogeneous nature of animal phenomenology. While based on markers clearly associated with consciousness in humans, most of these strategies are theoretical constructs lacking empirical data and are based on metrics appropriate for humans but difficult to measure in most non-human species. I propose a novel symbolic profile based on readily observable behaviors that logically constitute subjective experience across the entire spectrum of animals that possess a centralized nervous system. Three modes (markers) of behavior displayed by all animals – volition, interaction, and self-direction – are quantified according to the frequency, variety, and dynamism of each mode. The resulting matrix of 3 modes x 3 metrics can be expressed as a bi-directional heatmap, allowing for quick and easy inter-species comparisons. The overall effect is to highlight both similarities and differences in the subjective experience of animals ranging from crustaceans to primates.

## 1 Introduction

Acceptance of the view that consciousness is widespread, multimodal, and evolutionarily non-linear in complexity across the animal kingdom has increased in recent years (Chittka, 2017; Chittka and Wilson, 2019; Feinberg and Mallatt, 2016; Ginsburg and Jablonka, 2010; Irwin, 2020, 2024; Irwin et al., 2022). The assumption is growing that the subjective nature (phenomenology) of consciousness is variable across taxonomic clades, species-specific within clades, and developmentally dependent (Edelman, 1987; Kaufmann, 2021; Schnell and Clayton, 2020). Andrews (2024) has even advocated that consciousness in all animals in some measure should be a default presumption.

In concert with these trends, schemes for profiling different assumed patterns of consciousness in animals have begun to appear (Bayne et al., 2024; Birch et al., 2020; Dung and Newen, 2023; Pennartz et al., 2019). A great benefit of these proposed profiles has been to illustrate the way in which different dimensions of consciousness are likely manifested in different species. However, the profile schemes that have been proposed are nearly all theoretical constructs in the absence of actual data, and most are based on human mental experience and cognitive capacities (Andrews, 2024; De Waal, 2016), so their relevance to consciousness in non-human animals is largely conjectural – especially so with increasing evolutionary distance from humans. A greater advance would be provided by profiles based on empirical data. The study of comparative animal consciousness therefore is badly in need of metrics that can be assessed quantitatively across a large range of animal taxa.

At the same time, by its very nature, subjective experience in non-human animals is largely inaccessible due to the lack of complex, nuanced linguistic communication between human investigators and non-human subjects (Feinberg and Mallatt, 2018; Searle, 1992). Instead, one strategy being adopted increasingly is the study of proxy behaviors, or markers (Dung, 2022; Kaufmann, 2024), that, when exhibited by humans, are clearly associated with distinctive states of consciousness.

Because of the multiple definitions of consciousness, the various clinical uses of the term in medical and veterinary practice, and its philosophical history deeply embedded in the context of human mental experience, many authors have sought to use alternative terms such as “subjective experiencing” (Ginsburg and Jablonka, 2019), “phenomenal sensory experience” or “subjective feelings” (Bronfman et al., 2016), “subjective awareness” (John, 2003), “personal awareness” (Irwin, 2020), or “perceptual and affective experience” (Irwin et al., 2022). The term “experience” is present or implicit in most of these alternative definitions, and since ‘consciousness’ is a subjective (personal, or internally perceived) experience, “subjective experience” can be used as a synonym for consciousness without invoking necessarily the human version of it. Hence, that is the term that will be used in this paper.

My goal, first, is to propose some general modes of behavior that animals across all the major phyla exhibit and that can be quantified by simple observation. My second goal is to propose a simple symbolic representation of those quantified modes of behavior. This paper is an extension of the first use of this strategy (Irwin, 2024), but with added detail and emphasis on the methodology.

## 2 Methods

### 2.1 Subjects

Species were selected for study based on no set criteria other than phylogenetic diversity and the observer’s ability to view all three behavioral modes clearly but unobtrusively with quantitative precision. Most of the species providing data for this study were housed in a zoo or aquaria, which provide the advantage of making observations on a number of different animals from different clades under similar conditions observable in an efficient manner. Though the confinement of the animals arguably renders their behavior divergent to lesser or greater degrees from what it might be in their natural, unconstrained habitats, all the terrestrial species were housed in spacious naturalistic settings which enabled them ample opportunity for roaming about, interacting, or being solitary; and the aquatic species were confined to tanks large enough for free mobility.

### 2.2 Experimental design

**Figure 1:**
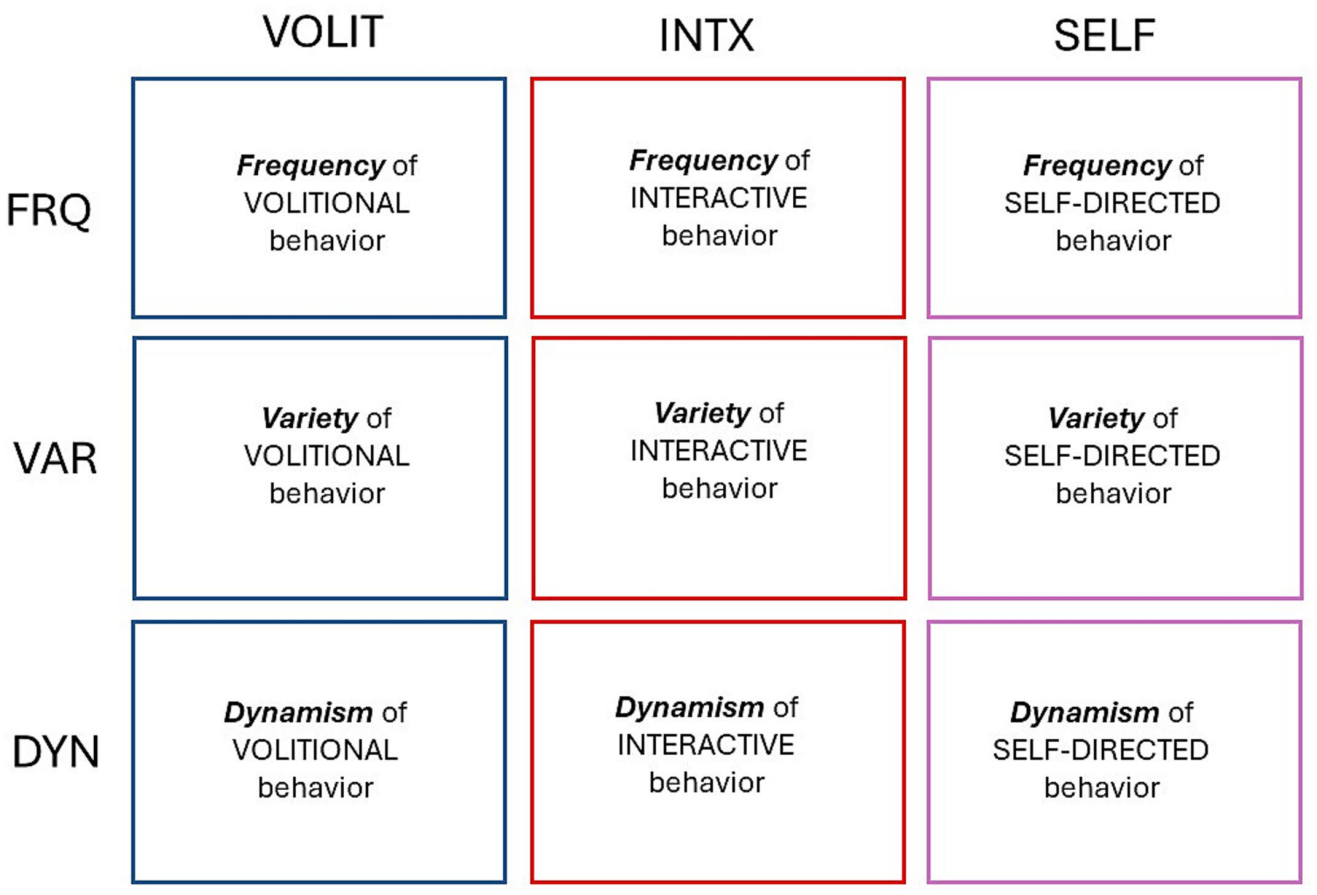
Bi-directional matrix for quantifying modes of behavior. Volitional (VOLIT), interactive (INTX), and self-directed (SELF) behaviors are quantified according to their frequency (FRQ), variety (VAR), and dynamism (DYN).

This was an observational study of spontaneous behavior in species across a broad range of the animal kingdom. The goal of the study was to focus on three modes of behavior that are easy to recognize in any animal, and to quantify each mode in three different ways. The behavioral modes selected for observation were **volitional, interactive,** and **self-directed.** The quantitative metrics were the *frequency,* var*iety,* and *dynamism* of each behavioral mode. The quantification of three modes of behavior with three metrics each generated a bi-directional matrix of nine behavioral attributes, or proxies for subjective experience in each species (Figure 1).

#### 2.2.1 Qualitative modes of behavior

Volitional behavior consisted of kinetic actions that appeared to be deliberative, intentional, and goal-directed. Generally, any movement of the subject from one place to another, or initiation of behavior that altered the subject’s condition or environment, was considered to be a unit of volitional behavior, provided the motion was clearly not a reflexive reaction to sudden stimuli. Lizards and chickens roaming about, elephants and komodo dragons diving into a pool of water, salamanders, seahorses, and cuttlefishes swimming from one place to another, monkeys jumping to another limb in a tree, and birds flying from one perch to another were examples of volitional behavior documented in this study. The relationship between volitional mobility and subjective awareness has previously been emphasized (Pennartz et al., 2019; Vallortigara, 2020).

Interactive behavior was defined as any contact, communication, aggression toward, defense against, engagement with, or reaction to a conspecific or allospecific individual. It included reaction to exteroceptive stimulation, like sounds or actions at a distance made by other individuals of the same or different species. Signaling behavior directed toward conspecific or allospecific individual, such as bobbing displays by lizards or courting displays by birds, were considered interactive. Grooming and other physical contacts with conspecifics was a frequent form of interaction observed in this study. Chasing, running or swimming away from, or incidentally contacting other individuals were also recorded as interactive behavior. While most interactive behavior entails volition, for the purposes of this study, interactive behavior was scored in a category of its own. Interaction with and analysis of external signals from other animals or the environment typically requires subjective awareness (Baumeister and Masicampo, 2010; Mellor, 2019).

Self-directed behavior entailed somatic attention to, awareness of, or use of the animal’s own body not otherwise scored as volitional or interactive behavior. A frequent example was self-grooming, but also included were yawning, scratching, licking, and washing. Not all such actions necessarily require subjective awareness, so only actions in alert animals that appeared to be initiated spontaneously in a non-reflexive manner were scored in this category. Any consumatory behavior, such as eating or drinking, was also scored as self-directed behavior. Egocentric behavior entails self-directed attention, and is presumed to imply a sense of self and often to reflect the animal’s affective state (Paul et al., 2020).

#### 2.2.2 Quantitative metrics for each behavioral mode

Three different measures for all three behavioral modes were quantified during each observational period, as follows.

*Frequency* was a measure of the proportion of an entire observational period during which each of the three behavioral modes were exhibited. The number of 60-s intervals during which the subject displayed volitional, interactive, and/or self-directed behavior was noted, and reported as a percentage of the total number of 60-s intervals comprising each observational episode.

*Variety* provided an indication of the range and heterogeneity of a subject’s behavioral repertoire. The variety of volitional behaviors was measured by the number of new and distinguishable deliberative actions taken. The variety of interactive behavior was quantified by the number of new and different encounters with or reaction to another individual of the same or different species. The variety of self-directed behaviors was quantified by the number of different forms of egocentric activity that were displayed during the observational period. Since these numbers were always much lower than data for the two other behavioral modes, they were multiplied by 10 to make their order of magnitude more comparable to that of the other two modes for graphic clarity.

*Dynamism* was a measure of how active the animal was, as measured by how frequently a new behavioral activity was initiated or a previously discontinued activity was resumed. Each mode of behavior could consist of one type of activity, with periodic starts and stops, or could entail changes from one type of activity to another. The number of times that activity within one mode of behavior was restarted after having been stopped previously, or that a new form of behavior was initiated, provided a quantitative measure of how dynamically each mode of behavior was exhibited. Dynamism provides an indication of the rate at which sensorymotor information is experienced by the animal.

### 2.3 Behavioral observations

Individual animals were scored by the same single observer at the zoo (around midday) or aquarium (late afternoon) in Denver, Colorado, United States, or in an open field of chickens on Kauai, Hawaii (mid-morning). Every behavioral variation observed within each mode was recorded for each 60 s interval within which it was observed by a distinctive indicator (e.g., for the macaw: h = hopping, v = vocalizing, g = self-grooming, x = reacting to sudden noise; for the elephant: a = walking about, w = washing, t = touching or feeling conspecifics, f = feeding). Each distinctive behavior was assigned to its relevant mode (e.g., hopping and walking were volitional movements; vocalizing and physical contact with conspecifics were scored as interactive behavior, while self-washing and feeding were recorded as self-directed actions.) Once segregated by mode, the attributes within each mode were quantified as described above.

**Figure 2:**
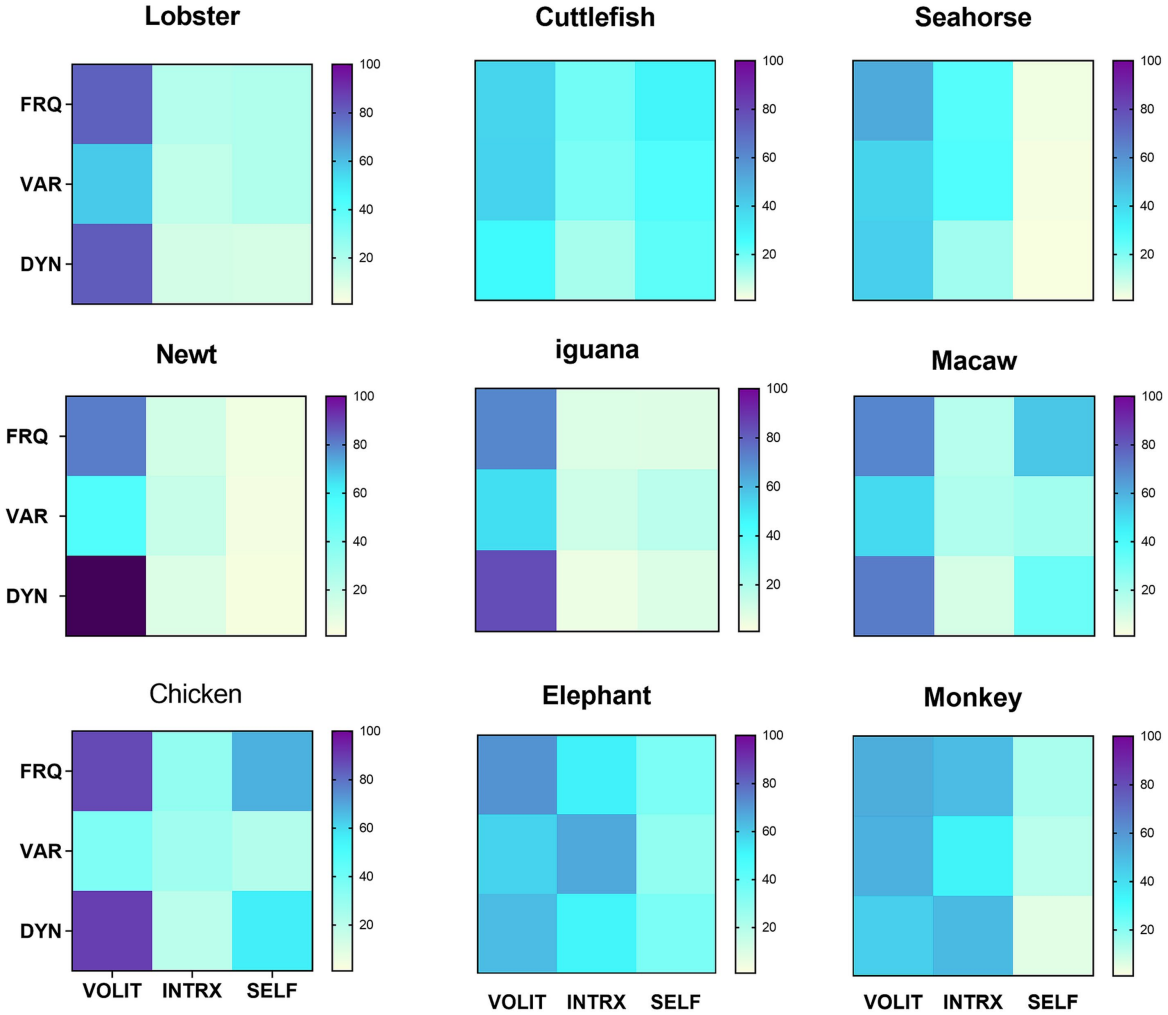
Symbolic representations of subjective experience, based on matrix profiles for nine species. Data are reported from arthropods (lobster), cephalopods (cuttlefish), bony fishes (seahorse), amphibians (newt), reptiles (iguana), birds (chicken and macaw) and mammals (elephant and monkey). Metrics were obtained as described in section 2.2.2. Values for variety were uniformly low, so were multiplied x10 for visual clarity. Mean CV = 34.8% for the frequency of volition in the nine species.

**Figure 3:**
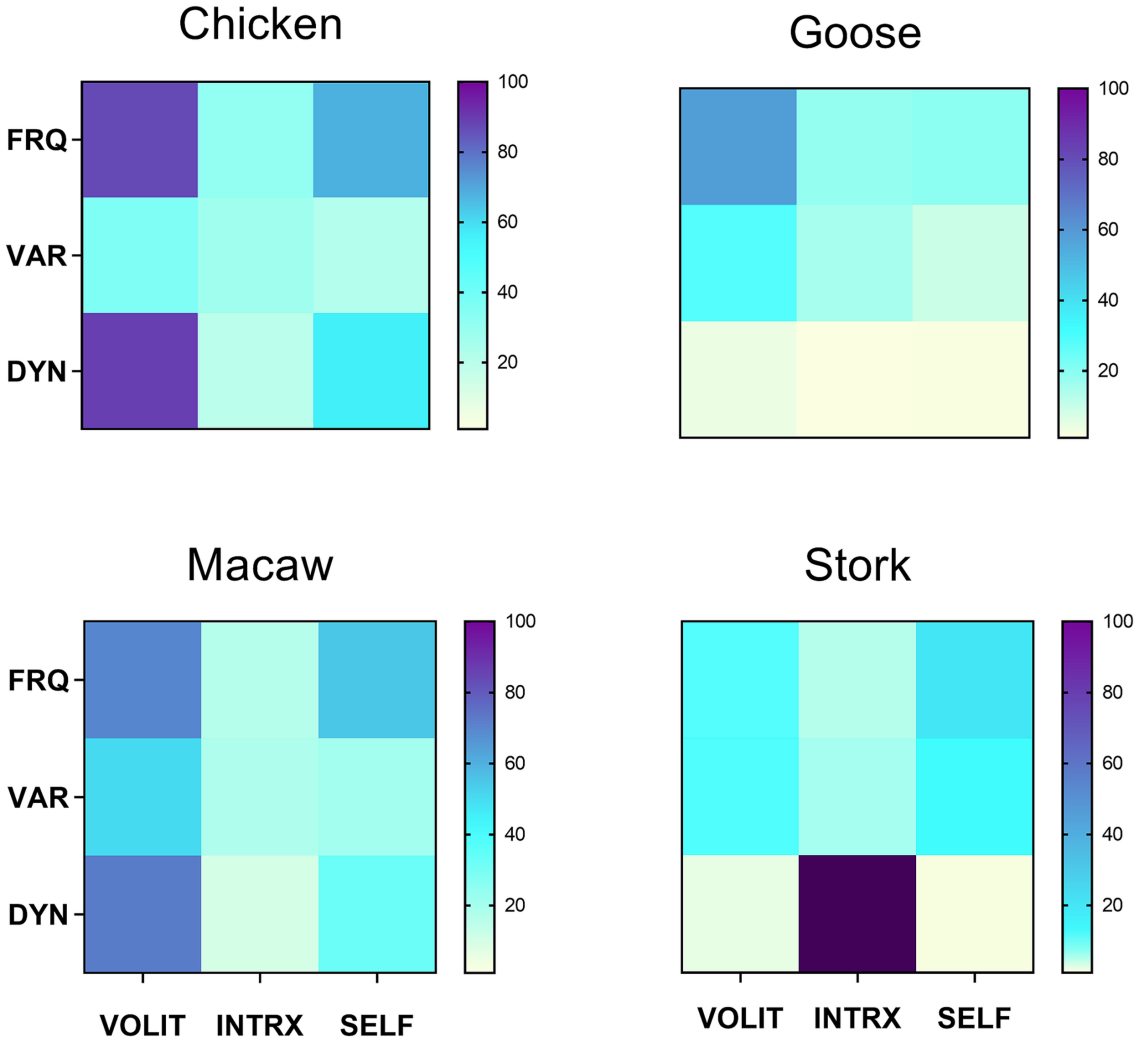
Symbolic representations of subjective experience, based on matrix profiles for four avian species. Metrics were obtained as described in section 2.2.2. Values for variety were uniformly low, so were multiplied x10 for visual clarity. Mean CV = 39.9 for the frequency of volition in the four species.

Having all data collected by the same observer precluded inter-observer variation. Trial observations of all potential species were made to ensure that behavioral modes and metrics could be identified without ambiguity within and across species. Coefficients of variation (CV) were computed for all samples to measure consistency of scoring within samples. The goal was to observe enough episodes for coefficients of variation (CV) in the frequency of volitional behaviors (the most reliably and consistently measurable attribute) for each species to be ≤40%. This was achieved with 4–6 separate observational episodes for all species with two exceptions: the cuttlefish (*n* = 6, CV = 64%) included in Figure 2, as the only available cephalopod representative, and the stork (*n* = 4, CV = 113%), added to Figure 3 to enlarge the range of avian species for comparison. Those species for which behavioral designations could not be made with confidence, or which raised the average CVs across all species in the comparisons in Figures 2, 3 above 40% (about a third of all those initially observed), were not included in those figures.

Most observations were 15 min in duration (range = 10–20 min), but the sum of all quantitative attributes in each session were normalized to 15 min for *frequency* and *interactions*, or 60 min for *dynamism* (to equalize the order of magnitude of *dynamism* with the other two modes for graphic clarity). Care was taken to keep observations unobtrusive with no evident influence on the behavior of the subjects. The distance between observer and subject was kept at the maximum feasible for accurate scoring. Different individual animals were scored for each observational episode whenever possible, and always on different days. The exceptions are noted in Table 1.

**Table 1:** Species, taxonomy, and location of observations.

Animal	*Species*	Taxonomy	Place observed
Caribbean Spiny Lobster	*Panulirus argus*	Arthropod/Crustacean	Denver Aquarium
Cuttlefish *	*Sepia* sp	Mollusk / Cephalopod	Denver Aquarium
Seahorse	*Hippocampus* sp.	Vertebrate / Bony fish	Denver Aquarium
Eastern Newt	*Notophthalmus viridescens*	Vertebrate / Amphibian / Urodele	Denver Zoo
Rhinocerus Iguana (F) *	*Cyclura ornata*	Vertebrate / Reptile / Squamata	Denver Zoo
Chicken (M)	*Gallus gallus*	Vertebrate / Aves / Galliformes	Open range in Kauai, Hawaii
Canadian Goose	*Branta canadensis*	Vertebrate / Aves / Anseriformes	Free ranging on open lawn in Denver, CO
Blue and Gold Macaw	*Ara arauna*	Vertebrate / Aves / Psittaciformes	Denver Zoo
Saddle-billed Stork	*Ephippiorhynchus senegalensis*	Vertebrate / Aves / Ciconiiformes	Denver Zoo
Reticulated Giraffe (F) *	*Giraffa camelopardolis*	Vertebrate / Mammal / Artiodactyl	Denver Zoo
Asian Elephant (M)	*Elephas maximus*	Vertebrate / Mammal / Proboscid	Denver Zoo
Black & White Colobus (M)	*Colobus guereza*	Vertebrate / Mammal / Primate	Denver Zoo

*The same individual was the subject of all observations.

When the sex of all subjects for a given species was the same for all observations, it is indicated by F for females and M for males in parentheses. The species with no sex designation were those in which each sex was observed in different sessions, or were not ascertainable.

### 2.4 Representation of behavioral profiles

The three modes of behavior, each quantified by three different metrics, generated a unique bi-directional profile of behavioral modes x quantitative metrics for each species. The resulting matrix of nine quantified behavioral attributes were expressed as heat diagrams in which each cell of the matrix was coded by the intensity of shading proportional to the average score for each mode x metric attribute.

The average magnitude of each score was computed as described in Section 2.2.2 above, except that any observational session with no occurrence of detectable interactive activity was not considered in calculating the average value for that attribute. Thus, zero values for interactive behavior were discounted, usually because of the absence of another animal that could provide the opportunity for interaction.

The primary objective of this study was to demonstrate how markers of presumed subjective experience can be compared across taxonomic units by contrasting visual representations of those patterns. A version of the Minkowski metric (Sinha and Russell, 2011) was used to quantify the degree of disparity between any two patterns. A difference index (DI) was calculated for every pairwise comparison, based on the Euclidean distance between the quantitative metrics for all nine attributes in one pattern (A) with all nine comparable attributes in another pattern (B), in which:


$$DI=\sqrt{\overset{n}{\underset{\alpha =1}{\sum }}{\left(A_{\alpha 1}-B_{\alpha 1}\right)}^{2}\ldots +{\left(A_{\alpha n}-B_{\alpha n}\right)}^{2}}$$

where *A* and *B* are the two patterns being compared,
*A_α1_* is the first attribute (frequency of volitional behavior) in pattern A.
*B_α1_* is the first attribute (frequency of volitional behavior) in pattern B.
*A_αn_* is the last attribute (dynamism of self-directed behavior) in pattern A.
*B_αn_* is the last attribute (dynamism of self-directed behavior) in pattern B.
A higher DI means a greater difference between the patterns being compared.
Data processing and graphing was carried out with GraphPad Prism, version 9.5.1.

## 3 Results

The heterogeneous and multimodal variation in behavioral profiles across the animal kingdom is apparent in the symbolic representations of those profiles (Figure 2). Volition was the most consistently observed and robust mode of behavior across all profiles, so quantitative data showing the means, standard deviations, sample sizes (which apply to all measures for that species), and coefficients of variation for that mode are given in Table 2. The ease of interspecies comparisons based on the visual patterns in Figure 2 compared to the jumble of numerical data in Table 2 is obvious. The appearance of similarity or dissimilarity of patterns between any given species with that of any other is backed up by the quantitative DI for pattern comparisons between any two species in the study (Table 3).

**Table 2:** Quantitative data on volitional behavior.

Species	Frequency	Variety	Dynamism
**Monkey**	**58 ± 18 (6) 33%**	**57 ± 10 (6) 18%**	**47 ± 14 (6) 30%**
**Elephant**	**74 ± 25 (6) 33%**	**60 ± 18 (6) 38%**	**60 ± 38 (6) 57%**
Giraffe	62 ± 35 (6) 56%	37 ± 20 (6) 54%	37 ± 24 (6) 65%
**Macaw**	**73 ± 23 (4) 32%**	**53 ± 22 (4) 42%**	**76 ± 58 (4) 76%**
**Chicken**	**89 ± 24 (5) 27%**	**32 ± 8 (5) 26%**	**91 ± 30 (5) 33%**
Goose	62 ± 8 (4) 13%	28 ± 13 (4) 46%	3 ± 2 (4) 46%
Stork	9 ± 10 (4)113%	10 ± 8 (4) 82%	2 ± 1 (4) 41%
**Lizard-Iguana**	**7 ± 19 (6) 27%**	**47 ± 19 (6) 40%**	**72 ± 35 (6) 49%**
Lizard-Earless	12 ± 6 (4) 48%	13 ± 5 (4) 40%	2 ± 1 (4) 55%
**Newt**	**83 ± 17 (6) 20%**	**54 ± 11 (6) 21%**	**130 ± 51 (6) 39%**
Toad	16 ± 10 (5) 67%	17 ± 12 (5) 69%	9 ± 6 (5) 65%
**Seahorse**	**53 ± 20 (6) 38%**	**38 ± 25 (6) 65%**	**41 ± 21 (6) 52%**
**Cuttlefish**	**42 ± 27 (6) 64%**	**42 ± 17 (6) 41%**	**28 ± 16 (6) 59%**
**Lobster**	**83 ± 32 (6) 38%**	**60 ± 21 (6) 35%**	**84 ± 45 (6) 54%**

Values are mean ± standard deviation with percent CV for (*n*) observational episodes. Species shown in boldface are included in Figure 2. The mean CV for those nine species was 34.8%.

**Table 3:** Degree of disparity* between all two-way comparisons.

	Lobster	Cuttlefish	Seahorse	Newt	Iguana	Macaw	Goose	Chicken	Stork	Giraffe	Elephant
Cuttlefish	73										
Seahorse	54	37									Difference
Newt	37	103	79								Index
Iguana	22	78	58	44							<40
Macaw	45	66	64	67	48						40–79
Goose	92	43	55	122	95	87					80–119
Chicken	77	97	95	86	78	46	116				120+
Stork	122	61	54	145	119	117	58	146			
Giraffe	71	41	57	100	71	47	50	77	82		
Elephant	89	94	130	106	96	85	118	87	148	99	
Monkey	78	103	53	100	88	86	91	106	114	88	59

*Calculated as described in Section 2.4. Larger numbers indicate a greater difference between the species being compared.

The pattern for many of the species reveals unique features, like extremely dynamic volitional behavior in the newt, the high frequency of self-directed behavior (mostly self-grooming) in the chicken, and the amount of variety in the interactive behavior among elephants (Figure 2). Beyond these unique features, though, some generalizations are possible. Volition is the most frequent and usually the most dynamic behavior seen across all subjects. This was particularly true of the smaller species, like the lobster, newt, and chicken. The avian and mammalian species scored higher on interactive behavior, while the seahorse and newt were almost totally lacking in self-directed behavior.

Behavioral profiles for four different avian species are shown in Figure 3, illustrating a range of behavioral patterns both similar and dissimilar. While the chicken and macaw have similar profiles (DI = 46), both differ much more from the patterns for the goose (DI = 87 vs. the macaw; DI = 116 vs. the chicken) and the stork (DI = 117 vs. the macaw; DI = 146 vs. the chicken).

## 4 Discussion

The main objective of this paper is to suggest a way to conceptualize differences in behavioral profiles for different animals, as a framework for the nature of phenomenology presumed to constitute subjective experience in different species. The heat diagrams are offered as symbolic representations of the presumed nature of subjective experience.

The data presented here reinforce the results of an earlier study in correlating similar patterns more closely with lifestyle than with habitat (Irwin, 2024). This is seen in similar profiles for the marine lobster, the freshwater newt, and the terrestrial iguana – all solitary foragers – while the monkey and elephant have quite different profiles though occupying similar habitats. A weak correlation with phylogeny can be seen in the simpler profiles of the fish (seahorse), amphibian (newt) and reptile (iguana), than the profiles for the avian and mammalian species, supported by generally larger DIs for comparisons between the former and the latter (Table 3).

The modes and metrics of markers for subjective experience studied here suggest a way of thinking about differences in the elements and complexity of animal behavior. They are not submitted as either measures or descriptors of phenomenology, but as empirical observations of behavioral patterns that in their similarities and differences suggest a variety of subjective experiences across species. They by no means represent the full range of behavior or the only aspects of experience that could point to the nature of animal minds. Grooming, object manipulation, vocalization or other forms of signaling (e.g., color changes or displays), territorial defense or aggression, gaze direction or duration, or courtship behavior could all serve as markers for different forms of subjective experience. Any of these or other modes of behavior that could be quantified in different ways could be used to generate a matrix like the ones presented here.

A potential criticism of this study is that the definitions for the three modes of behavior are imprecise and call for subjective judgments by the observer. Descriptions of behavior general enough to cover species over a large range of the phylogenetic spectrum are necessarily less precise than when a single species is under investigation. It is not logical to assume, for instance, that volitional or interactive behaviors are going to be precisely definable in the same way between monkeys and lobsters. However, that does not compromise the value of the data reported here in the context of the study’s objectives. Note that the focus of the methodology proposed is on the pattern of the qualitative x quantitative mosaic of subjective experience across species, however that experience is operationally defined. That is, the specific boundaries of each mode (as defined consistently, even if subjectively, by the observer) are less critical than the quantitative features of that mode in relation to the quantitative measures for that mode in other species. Consistency of observation is more important than the supposed variance in scoring by different observers. That observational consistency was achieved in this study is supported by a CV < 40% for nine of the 12 subject species.

**Figure 4:**
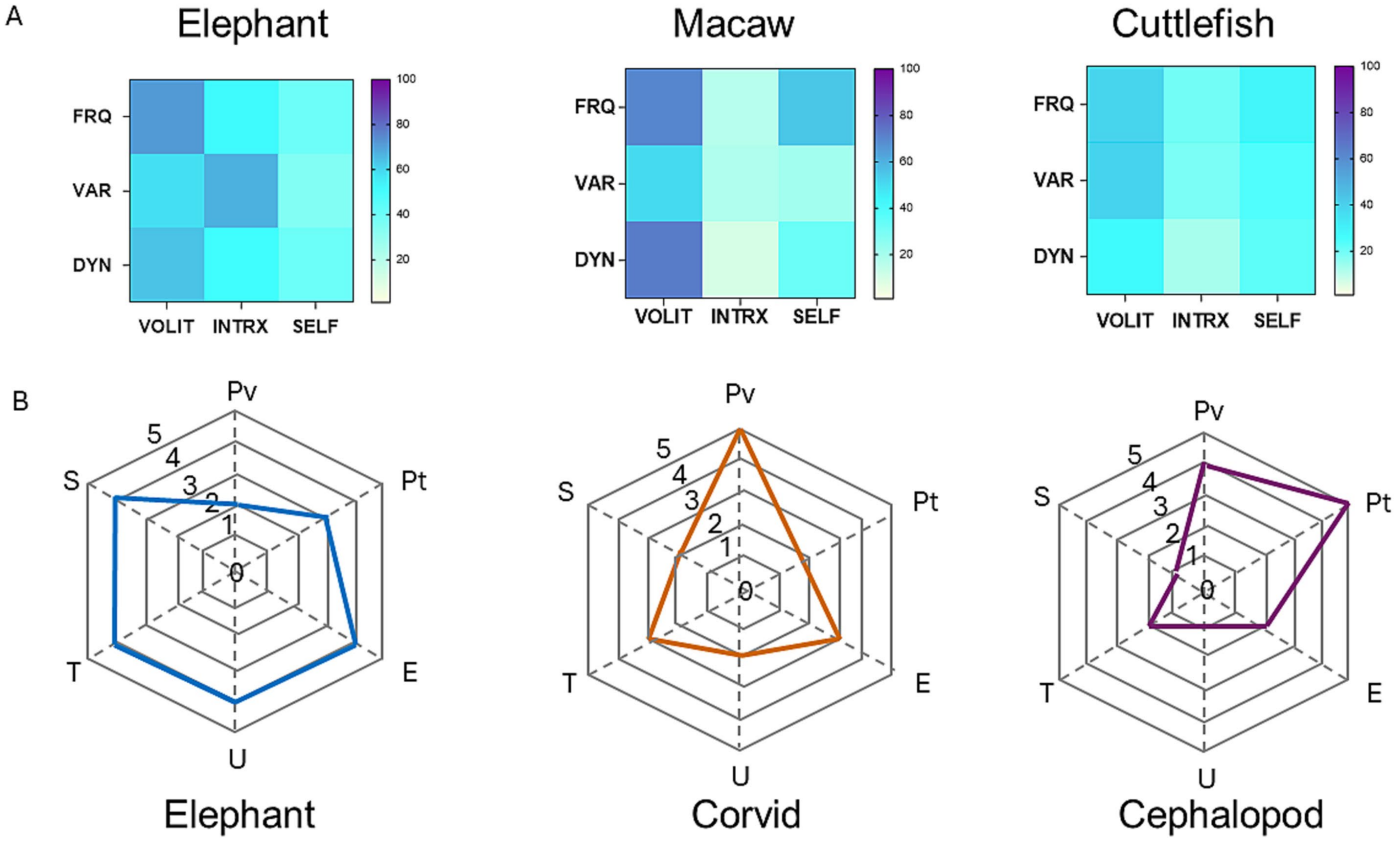
Comparison of two methods for the representation of behavioral profiles and cognitive dimensions in elephants, birds, and cephalopods. **(A)** Heat maps constructed by the methods described in this study based on empirical behavioral observations of elephants, macaws, and cephalopods (cuttlefishes). **(B)** Hypothetical representations based on theoretical estimates of six cognitive attributes in generic elephants, corvids, and cephalopods. Redrawn from data provided in Birch et al. (2020). Abbreviations: U, unity; T, temporality; S, selfhood; Pv, perceptual richness of vision; Pt, perceptual richness of touch; E, evaluative richness.

Using a similar strategy, Birch et al. (2020) proposed that consciousness in any species could be represented as a polygon formed by connecting six points corresponding to different aspects of mental experience, each differing in magnitude in a species-specific manner. A comparison of their method, invoking theoretical expectations of the magnitude of each mode in an elephant, corvid, and cephalopod is compared with empirical data from this study on elephants, a macaw, and a cephalopod (cuttlefish) in Figure 4. It is interesting to note that the areas of the polygons generated in the study by Birch et al. (2020) bear a rough parallel with the complexity of the heat diagrams based on actual data in this study.

**Figure 5:**
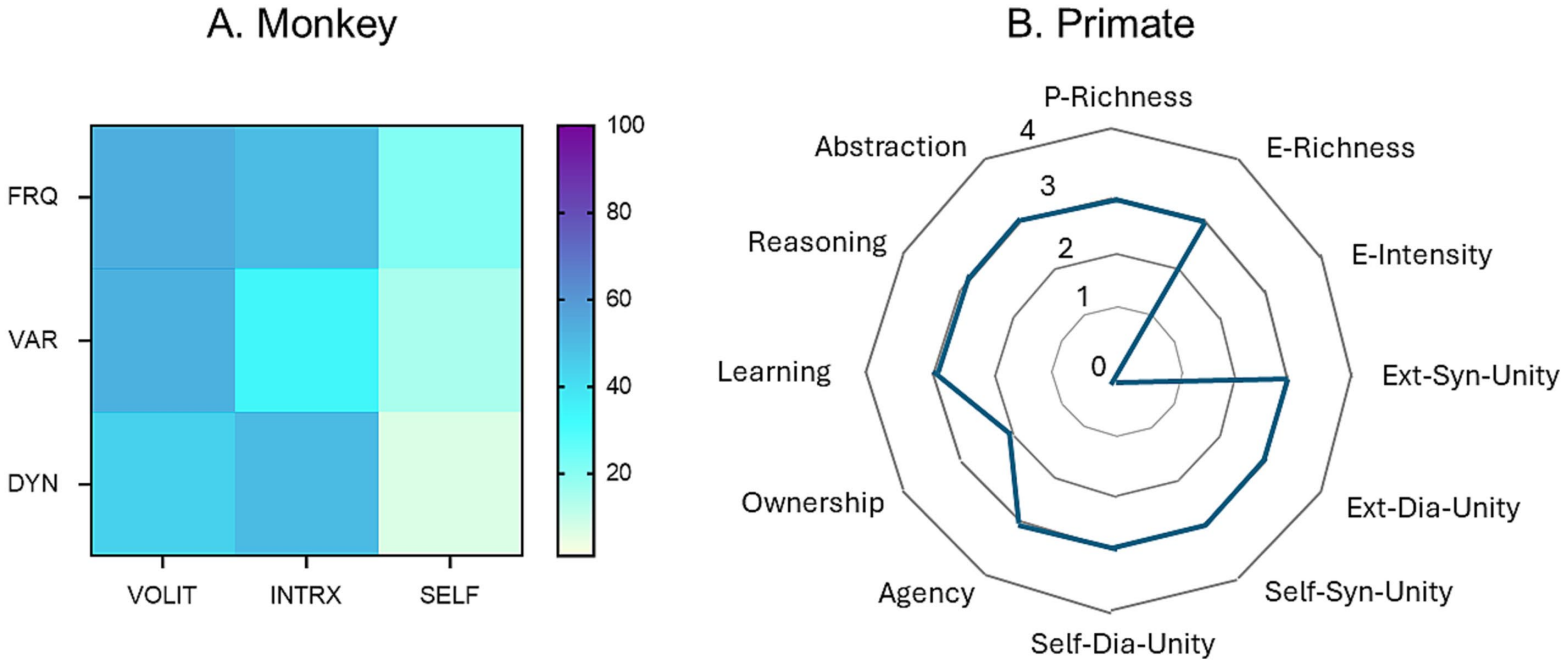
Comparison of two methods for the representation of behavioral profiles and cognitive dimensions in primates. **(A)** Heat map constructed by the methods described in this paper based on empirical behavioral observations of a monkey. **(B)** Hypothetical representations based on theoretical estimates of degrees of expression of 10 dimensions of consciousness in a generic primate. Redrawn from data provided in Dung and Newen (2023). Abbreviations: P, perceptual; E, evaluative; Ext, external; Syn, synchronic (occurring at one point in time); Dia, diachronic (occurring across time).

A related but different strategy was proposed by Dung and Newen (2023). They expanded the putative markers for consciousness to 12 different attributes, connected by lines forming a polygon on a four-dimensional scale based on assumed magnitudes of absent (0), weakly present (1), moderately present (2), or strongly present (3) estimations for each attribute. A comparison between their profile for generic primates and the empirical data for monkey reported in this study is shown in Figure 5.

All three symbolic representations of behavioral profiles rely on markers that logical extension from comparable human experience indicates are most likely to be coextensive with subjective experience in animals. The variety seen in all three representations are consistent with the view that there is no single scale along which experiential complexity varies (Birch et al., 2020; Irwin, 2020; Kaufmann, 2021). Rather, the multiple patterns of behavior seen across different taxa reflect the variety of species-specific lifestyles, adaptations, and specializations that have diversified through evolutionary history. For example, the active chicken, elephant, and monkey all display complex but distinctive profiles, while the cuttlefish and seahorse, distantly related from each other, show similar profiles to one another consistent with their aquatic lifestyles. The behavioral profile of the goose suggests a simpler cognitive repertoire than that of the chicken and macaw (Figure 3). These are merely examples of the types of inferences that can be derived by cross-species comparisons of the symbolic representations suggested here. Other investigators, applying their expertise with other markers and metrics, should be able to derive further insights into comparative animal consciousness using this strategy.

## 5 Conclusion

A set of three behavioral modes, each quantified in three different ways, is proposed as a means of generating a unique behavioral profile for interspecies comparisons across the animal kingdom. All three modes clearly coincide with human phenomenology, thus are suggested as proxies for subjective experience by all animals with complex (hierarchically organized) nervous systems, in any taxonomic category. The data reported in this study and its predecessor (Irwin, 2024) indicate a range of variability and complexity consistent with the view that all animals are indeed “conscious in their own way” (Kaufmann, 2024).

## Data Availability

The raw data supporting the conclusions of this article will be made available by the authors, without undue reservation.
